# Well Defined Poly(Methyl Methacrylate)-Fe_3_O_4_/Poly(Vinyl Pivalate) Core–Shell Superparamagnetic Nanoparticles: Design and Evaluation of In Vitro Cytotoxicity Activity Against Cancer Cells

**DOI:** 10.3390/polym12122868

**Published:** 2020-11-30

**Authors:** Graciane Resende, Gabriel V. S. Dutra, Maria S. B. Neta, Olacir A. Araújo, Sacha B. Chaves, Fabricio Machado

**Affiliations:** 1 Laboratório de Desenvolvimento de Processos Químicos, Instituto de Química, Universidade de Brasília, Campus Universitário Darcy Ribeiro, CEP 70910-900 Brasília, DF, Brazil; graciane.resende@aluno.unb.br (G.R.); gabriel.victor@aluno.unb.br (G.V.S.D.); 2 Departamento de Genética e Morfologia, Instituto de Biologia, Universidade de Brasília, Campus Universitário Darcy Ribeiro, CEP 70910-900 Brasília, DF, Brazil; sousa.brito@aluno.unb.br (M.S.B.N.); sachabraun@unb.br (S.B.C.); 3 Universidade Estadual de Goiás, Campus Central—Ciências Exatas e Tecnológicas, CP 459, CEP 75132-903 Anápolis, GO, Brazil; olacir.araujo@ueg.br

**Keywords:** methyl methacrylate, vinyl pivalate, magnetic nanoparticles, embolization, miniemulsion, seeded emulsion, core–shell polymer particles

## Abstract

The objective of this work is to develop and characterize polymeric nanoparticles with core–shell morphology through miniemulsion polymerization combined with seeded emulsion polymerization, aiming at the application in the treatment of vascular tumors via intravascular embolization. The synthesis of the core–shell nanocomposites was divided into two main steps: (*i*) Formation of the core structure, consisting of poly(methyl methacrylate)/magnetic oxide coated with oleic acid (OM-OA) via miniemulsion and (*ii*) shell structure produced through seeded emulsion polymerization of vinyl pivalate. Nanocomposites containing about 8 wt.% of OM-OA showed high colloidal stability, mean diameter of 216.8 nm, spherical morphology, saturation magnetization (M_s_) of 4.65 emu·g^−1^ (57.41 emu·g^−1^ of Fe_3_O_4_), preserved superparamagnetic behavior and glass transition temperature (*T*_g_) of 111.8 °C. TEM micrographs confirmed the obtaining of uniformly dispersed magnetic nanoparticles in the PMMA and that the core–shell structure was obtained by seeded emulsion with M_s_ of 1.35 emu·g^−1^ (56.25 emu·g^−1^ of Fe_3_O_4_) and *T*_g_ of 114.7 °C. In vitro cytotoxicity assays against murine tumor of melanoma (B16F10) and human Keratinocytes (HaCaT) cell lines were carried out showing that the core–shell magnetic polymeric materials (a core, consisting of poly(methyl methacrylate)/Fe_3_O_4_ and, a shell, formed by poly(vinyl pivalate)) presented high cell viabilities for both murine melanoma tumor cell lines, B16F10, and human keratinocyte cells, HaCaT.

## 1. Introduction

The development of polymeric materials is undoubtedly attractive in the manufacture of many biomedical devices, given their structural versatility, ease of manufacture, biocompatibility, low cytotoxicity, flexibility, biodegrability, porosity, and controlled morphology, among others [1,2]. In this field, highlight the nanocomposites that combine the thermoplastic properties of polymers and the superparamagnetic behavior of nanoparticles, presenting great potential in the treatment of vascular tumors [3,4].

Iron oxide nanoparticles possessing various characteristics such as biocompatibility, superparamagnetic properties, and recyclability [5] have been widely used in controlled drug delivery systems, facilitating drug channeling to specific target cells by externally controlling their path using a field [6], and as a contrast agent for magnetic resonance, hyperthermia treatment of tumors and detoxification of biological fluids [7,8,9], etc. 

In biomedical applications, the encapsulation of nanoparticles with polymeric matrices ensures their stability in biological media, provides functionality, protects and prevents agglomeration [10] and results in increased local drug concentrations, enabling their use for targeted delivery of therapeutic agents, reducing toxic effects in cells or tissues healthy [11,12]. The polymeric matrices can be prepared from synthetic or natural polymers, obtaining colloidal particles by several synthetic routes, including emulsion [13,14,15], suspension [16,17,18], and miniemulsion polymerization [19,20,21].

Embolization therapy involves deliberate blocking of blood vessels due to the introduction of embolic agents, with the aim of decreasing or preventing blood transport through the blood vessels towards the injured area [22]. The choice of the adequate embolizing agent is important for the safety and effectiveness of the treatment [23]. Embolic agents can be classified into two main categories: Temporary and permanent. Although temporary agents block blood flow, they will be dissolved or eliminated by the immune system within a prescribed period of time. For the temporary embolic materials, the most used materials are autologous agents and resorbable gelatin sponge. Permanent agents are designed to provide irreversible blood flow obstruction to target lesions or final organs. In particular for the permanent embolic agent, the most used materials are coils, plugs, detachable balloons (solid agents), polyvinyl alcohol, compressible microspheres, thrombin, dehydrated alcohol, sodium tetradecyl sulfate, glues, and onyx (liquid agents) [23,24].

The miniemulsion polymerization process has excelled in recent years due to the intrinsic characteristics of the materials obtained, such as narrow particle size distributions, high colloidal stability, high molar mass, obtaining a wide range of polymers and mainly, the ability to encapsulate materials inorganics in a single reaction step with fast polymerization rates [25,26,27,28]. A versatile class of nanocomposites can be developed by combining the peculiarities of the miniemulsion with other polymerization techniques, obtaining polymeric nanoparticles of the core–shell type, the core being produced from the polymer initially polymerized, and the polymers formed later are incorporated into the layer external [29]. These materials have final properties dependent not only on the composition of each polymeric phase, but also on the morphology of these particles [30].

Core–shell polymer nanoparticles have attracted great interest due to their unique structure in which two different components are combined into a single material [31]. In biological applications, core–shell nanoparticles have great advantages over conventional nanoparticles, thus improving performance, such as low cytotoxicity, good dispersibility, high biocompatibility and cell compatibility, better connection with other biologically active molecules, as well as improved thermal and chemical stability [6,32,33].

With this perspective, Rahman et al. [34] developed a thermo-responsive magnetic polymer, synthesizing the magnetic seeds of cross-linked polystyrene by emulsion polymerization. The thermosensitive poly(*N*-isopropylacrylamide) shell (PNIPAM) was obtained via precipitation polymerization and then the polymer was functionalized with aminoethylmethacrylate hydrochloride (AEMH). The TEM micrographs confirmed the core–shell morphology of the particles. Chen et al. [35] synthesized hybrid epoxy-acrylic latex with core–shell morphology, using an epoxy dispersion as seed and, subsequently, carried out the seeded emulsion polymerization in acrylic monomer. Recently, Li et al. [36] synthesized cross-linked polystyrene nanospheres via dispersion polymerization and, subsequently, these seeds were swollen in tert-butyl acrylate and styrene monomers and polymerized by seeded emulsion. The authors investigated the effect of methanol concentration, used in the synthesis of seeds, and the degree of crosslinking on the morphology of the nanoparticles.

For the synthesis of core–shell nanoparticles to be successful, the polymerization technique and the operating conditions used are highly fundamental [37]. In the current work, the technique used for the formation of the shell is the seeded emulsion, which consists of the formation of a new polymer layer grafted on the seed surface (N.B. herein, the core nanoparticles consisting of poly(methyl methacrylate)/Fe_3_O_4_ is synthesized through miniemulsion polymerization process), through addition of fresh monomer feed following a semibatch mode operation, leading to the formation of a uniform shell and promoting easy control of final core–shell morphology [38,39].

The focus of this work is to obtain core–shell particles through combination of a batch mode (used to form the core structure via miniemulsion polymerization) and a semicontinuous mode operation (to form the particle shell structure through seeded emulsion polymerization), in which the feed composition is adequately controlled, allowing for the formation of a new polymer phase on the previously formed polymer particle. These particular core–shell polymer particles, intended for to be used as an embolic agent, consist basically of *(i)* a core, consisting of oleic acid functionalized magnetic nanoparticles (OM-OA), properly dispersed into spherical poly(methyl methacrylate) nanoparticles (PMMA), and *(ii)* a shell, consisting of poly(vinyl pivalate) (PVPi). By the combination of vinyl pivalate and methyl methacrylate, this new magnetic embolic agent based on PVPi/PMMA exhibits low susceptibility to undesirable hydrolysis reactions, which is strongly responsible for the recanalization of blood vessels, since they are minimized by the steric effect caused by the insertion of PVPi into the particles due to the tert-butyl group present in its chemical structure [17,40]. In addition, these nanocomposites have good chemical and mechanical resistance and high thermal stability, conferred to the methyl methacrylate based core [41]. As an additional study, the cytotoxicity of PVPi/PMMA core–shell particles was evaluated in vitro for tumoral cell lines B16F10 (murine melanoma) and HaCat (human keratinocyte). In vitro studies are of fundamental importance to give insight into the cytotoxicity ability of the magnetic nanocomposites on tumoral and non-tumoral cell lines. In the current work, B16F10 and HaCat were selected because murine B16F10 melanoma model is widely used as metastatic melanoma model for preclinical studies, as well as, human keratinocyte HaCaT cell line model is extensively used in epidermal homeostasis and pathophysiology studies.

## 2. Materials and Methods

**Scheme 1:**
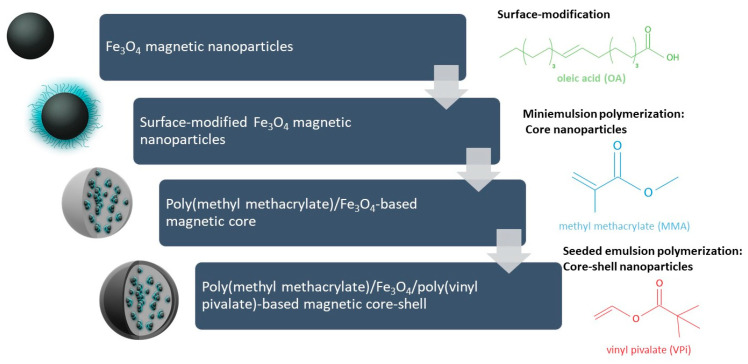
Flowchart summarizing the experimental protocol for formation of poly(methyl methacrylate)/Fe_3_O_4_/poly(vinyl pivalate) magnetic core–shell nanoparticles.

All reagents were analytical grade (PA) and used without prior purification. Methyl methacrylate (MMA) 99%, vinyl pivalate (VPi) 99%, hexadecane 99%, benzoyl peroxide 75% and ethylene glycol dimethylacrylate (EGDMA) 98% were purchased from Sigma-Aldrich Brasil Ltda. (Rio de Janeiro, Brazil). Hydrochloric acid (HCl) 36.5–38% and oleic acid 99% were purchased from Synth (São Paulo, Brazil). Ferric chloride hexahydrate (FeCl_3_·6H_2_O) 97%, ferrous sulfate heptahydrate (FeSO_4_·7H_2_O) 99%, sodium hydroxide (NaOH) 99% and potassium persulfate (KPS) 99% were purchased from Vetec Química Fina Ltda. (Rio de Janeiro, Brazil) and 90% pure sodium dodecyl sulfate (SDS) was supplied by Reagen Produtos para Laboratórios Ltda. (Parana, Brazil). Nitrogen gas was supplied by White Martins Gases Industriais Ltda. (Rio de Janeiro, Brazil) with 99.5% purity. Scheme 1 illustrates the experimental protocol for the synthesis of poly(methyl methacrylate)/Fe_3_O_4_/poly(vinyl pivalate) magnetic core–shell nanoparticles.

### 2.1. Synthesis of Magnetic Nanoparticles (OM)

Magnetic nanoparticles (OM) were obtained by the alkaline hydrolysis coprecipitation method of Fe^2+^ and Fe^3+^ ions in watery environment, as described by Neto et al. [42] in 500 mL of distilled water were added 2 mL of a 10% HCl solution and 0.03 mol of Fe^2+^ and Fe^3+^ metal ions in a stoichiometric molar ratio of 1:2 with 0.01 mol of FeSO_4_·7H_2_O and 0.02 mol of FeCl_3_·6H_2_O. The mixture was heated to 70 °C under constant stirring at 500 rpm and under nitrogen atmosphere for 30 min. Then the precipitating agent, NaOH, with a concentration of 5 mol L^−1^ was added until the solution reached a pH of approximately 12. After precipitation, the suspension remained under stirring at 70 °C and bubbling nitrogen for 30 min. The precipitate was washed with distilled water and decanted with the aid of a magnet until it reached neutral pH.

### 2.2. Coating of Magnetic Nanoparticles with Oleic Acid (OM-OA)

The magnetic nanoparticles were suspended in 170 mL of distilled water and then subjected to mechanical stirring under nitrogen bubbling and 85 °C heating. Then the coating material, 2.5 mL oleic acid, was added dropwise at a maximum rate of 0.5 mL min^−1^. After 30 min of the addition of oleic acid, the nanoparticles were washed with distilled water to neutral pH and treated with acetone to remove free coating material [16,42].

### 2.3. Synthesis of PMMA/OM-OA Magnetic Nanocomposite

For the production of nanocomposites, different parameters such as temperature, reaction time, amount of initiator and OM-OA content were studied. The concentration of surfactant, SDS, used in the synthesis was below the critical micellar concentration (CMC) at a temperature of 85 °C, which corresponds to 4.35 g L^−1^ [43] and 3.68 g L^−1^ at 70 °C (N.B. different from emulsion polymerization process, the amount of surfactant is kept below CMC in order to avoid the formation of micelles in the continuous aqueous phase, which guarantees that the polymerization reactions take place preferably inside the monomer nanodroplets).

The nanocomposite was synthesized by miniemulsion polymerization as follows: In one case the aqueous phase was homogenized with 153 g of distilled water and 0.6 g of SDS under constant stirring at 300 rpm for 10 min. Separately, the organic phase consisting of 27 g MMA, 0.81 g hexadecane, 0.72 g BPO and 4.05 g OM-OA was prepared in a beaker and homogenized using an ultrasonic disperser (Cole-Parmer^®^ 750 W, Vernon Hills, IL, USA) for 5 min at 40% amplitude in an ice bath. Subsequently, the organic phase was mixed into the aqueous phase and kept under constant stirring at 2700 rpm for 20 min in a mechanical disperser (IKA T25 digital ULTRA TURRAX^®^, Wilmington, NC, USA). Then the mixture was homogenized again through the ultrasonic disperser for 4 min at 70% amplitude in an ice bath. Finally, the mixture was added to a jacketed reactor at 85 °C and maintained by constant stirring at 400 rpm for 2 h.

### 2.4. Production of Poly(Vinyl Pivalate) Shell via Seeded Emulsion Polymerization

Core–shell morphology nanoparticles (PMMA/OM-OA/PVPi) were obtained using the seeded emulsion method [44], where PMMA/OM-OA nanocomposite seeds were fed with vinyl pivalate (VPi), initiator (KPS), and cross-linking agent (EGDMA) for shell formation. In a 500 mL tritubulated flask was added 200 g of PMMA/OM-OA colloidal dispersion (N.B. mass fraction of PMMA/OM-OA nanoparticles equal to 13.6%). The main components of the seeded emulsion polymerization step were initially separated into two phases, added separately into the PMMA/OM-AO colloidal dispersion: *(i)* The organic phase consisting of approximately 5.155 g VPi and 0.103 g EGDMA and *(ii)* the aqueous phase 20.5 g distilled water, 0.12 g SDS and 0.103 g KPS. The PMMA/OM-OA colloidal dispersion was kept under constant agitation at 500 rpm at 70 °C. Organic phase feeding was performed using a Solenoid Metering pump (ProMinent^®^, Gala series gamma/L 1000, São Paulo, Brazil) with a rate of 2.56 g h^−1^. After 1h of feeding the organic phase, the aqueous phase was slowly added for about 1 h. Then the remainder of the organic phase was added at the same rate. Subsequently, the polymerization was maintained for a further 2 h at 70 °C in inert nitrogen atmosphere.

### 2.5. Sample Characterization

X-ray diffraction measurements were used to identify the crystallographic phases present in the dimensions and the average size of the crystals. The diffractograms were acquired using a Rigaku-D/MAX X-Ray diffractometer (Austin, TX, USA), with CuKα (λ = 1.542510 Å) as a radiation source and a Ni-filtrate with CBO monochromator operating at 35 kV and 15 mA. The measurements performed in the variation range of 25° ≤ 2θ ≤ 80° with an increment of 0.05° and angular velocity of 1.0° min^−1^.

The magnetization measurements were obtained using a vibrating sample magnetometer (VSM, MicroSense, Lowell, MA, USA), ADE Magnetics model EV-9, with a magnetic field from −18 to +18 (kOe), at room temperature.

Energy dispersive X-ray spectroscopy (EDX) analyses measurements were carried out on an EDX-720 fluorescence spectrometer (Shimadzu Europa GmbH, Duisburg, Germany). EDX spectra were acquired under vacuum with a 10 mm collimator at accelerating voltage of 50 keV with steps of 0.02 and a live time of 100 s, using the Ti-U channel.

Fourier transform infrared spectra (FTIR) were displayed by dispersing bottles in KBr and presented as pellets. The spectra were acquired on a Varian Model 640 spectrometer (Varian Instruments, Santa Clara, CA, USA) with a resolution of 4 cm^−1^, updating 16 scans per sample.

The average diameter of monomer droplets (Dg) and polymeric particles (Dp) were determined using the dynamic light scattering technique (DLS) using a Malvern model Zetasizer Nano ZS (Malvern Instruments Ltd, Malvern, UK). For the analyzes, the samples were diluted with distilled water.

Transmission electron micrographs (TEM) were obtained using a JEOL JEM-2100 (Jeol Ltd., Tokyo, Japan) microscope operated at 200 kV. The samples were dispersed in water and sonicated for 5 min, where a drop of the suspension was applied on a copper screen containing a 200 mesh supported carbon film and dried at room temperature. The differential scanning calorimetry (DSC) curves were obtained using a Shimadzu equipment (model DSC-60, Shimadzu Scientific Instruments, Columbia, MD, USA). The initial masses used were approximately 5.0 mg and aluminum crucibles were used. For analysis, two heating ramps were performed, using a temperature range of −30 to 180 °C, with a temperature ramp of 10 °C·min^−1^_,_ He flow of 30 mL·min^-1^ and cooling was performed using liquid N_2_. The second heating ramp was used to determine the glass transition temperature (*T*_g_) of the nanocomposites.

The conversions were determined by gravimetry, and the latexes were added in previously weighed aluminum foil capsules, treated with 2 g·L^−1^ hydroquinone solution and dried in an oven at 60 °C for approximately 48 h. The conversion was obtained by the ratio of the dry polymer mass to the monomer mass used.

### 2.6. In Vitro Cytotoxicity Assays

The polymer materials were evaluated through in vitro cytotoxicity tests with murine melanoma (B16F10) and human keratinocyte (HaCat) tumor lines provided by Cell Bank of Rio de Janeiro, Brazil and maintained in culture at Nanobiotechnology Laboratory of the University of Brasília, UnB. All cell lines were cultured in DMEM (Dulbecco’s Modified Eagle Medium, provided by Sigma-Aldrich Brasil, São Paulo, Brazil) medium buffered with sodium bicarbonate and supplemented with 1% antibiotic (100 IU·mL^−1^ penicillin and 100 µg·mL^−1^ streptomycin) and 10% fetal bovine serum (FBS) at pH 7.2. Cells were maintained in cell culture flasks into an incubator at 37 °C, under 95% humidified air and 5% CO_2_ atmosphere, and the assays were performed in the logarithmic growth phase of the cells. Viable cells were quantified by using 40 µL of solution of Trypan Blue dye added in 10 microliters of cell suspension. The samples were evaluated for 24 h, with concentrations equal to 0.01 mg·mL^−1^, 0.05 mg·mL^−1^, 0.1 mg·mL^−1^, 0.25 mg·mL^−1^, and 0.5 mg·mL^−1^. The tumor cell lines (B16F10 and HaCat) were seeded in 96-well plates at a concentration of 2·10^3^ and 5·10^3^ cells per well with DMEM medium. Cell viability was determined by using the 3-(4,5-dimethylthiazol-2-yl)-2,5-diphenyl tetrazolium bromide-reducing colorimetric method (MTT) through spectrophotometry measurements using a 595 nm wavelength, whose results correspond to an average of three independent experiments in triplicates.

## 3. Results and Discussion

**Figure 1:**
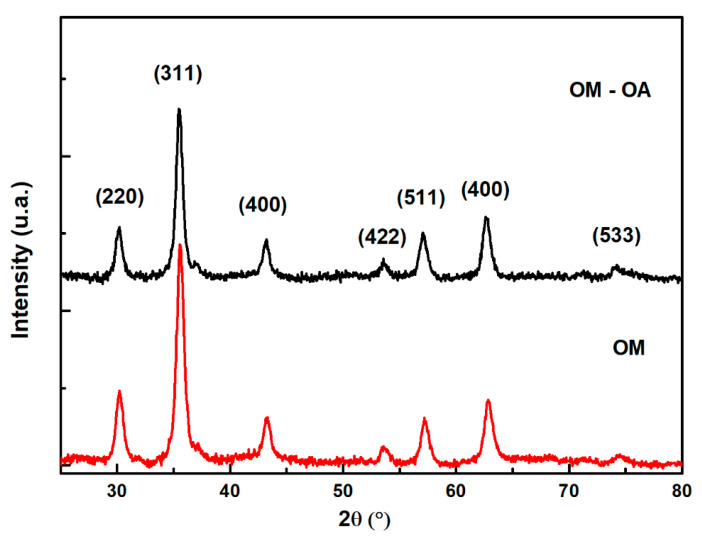
X-ray diffractograms of samples of magnetic oxide (OM) and oleic acid functionalized magnetic oxide (OM-OA).

Figure 1 shows the powder X-ray diffractograms and their diffraction patterns of OM and OM-OA samples. All peaks were analyzed and compared using data from the Joint Committee on Powder Diffraction Standards (JCPDS). The characteristic peaks of magnetic particles were observed at 2θ = 30.2°, 35.6°, 43.3°, 53.6°, 57.3°, 62.8°, and 74.5°, respectively, corresponding to the reflections of the crystalline planes (220), (311), (400), (422), (511), (440), and (533) of typical magnetite and/or maghemite spinel type cubic crystal structure. The diffraction patterns of the samples were similar, showing that the presence of oleic acid did not influence the structural and crystalline properties of nanoparticles [45].

The identification of the magnetite (Fe_3_O_4_) and maghemite (γ-Fe_2_O_3_) phases by XRD is quite complex, as both have the same spinel-like cubic crystal structure and therefore exhibit similar diffraction patterns. One way to determine the predominant crystalline phase is to use the peak position corresponding to the crystalline plane (440), with a value of 62.5° for the magnetite phase and 62.9° for maghemite [46]. The samples synthesized here have this peak located at 62.8°, indicating the presence of both phases.

The mean crystallite size (*D_DRX_*) was calculated from the width at half height of the highest intensity peak (FWHM) from the Scherrer equation (Equation (1)).

(1) $$D_{DRX}=\frac{0.9\lambda }{\beta \;cos\;\theta }$$

where: 0.9 is the proportionality constant for the spherical shape of the particles; *λ* is the wavelength of radiation CuKα (1.54056 Å); *β* is the half-height peak width corrected by the standard sample; *θ* is the Bragg diffraction angle of the most intense peak relative to the reflection of the crystalline plane (311).

The average crystallite sizes obtained were 10.5 nm (OM) and 12.2 nm (OM-OA), this increase was also observed in other studies [47,48,49]. These values are important from the point of view of application in intravascular embolization, since the superparamagnetic character is essential for this medical procedure and is characteristic of particles with an average size smaller than the critical size, generally agreed to be less than 20 nm [50].

**Figure 2:**
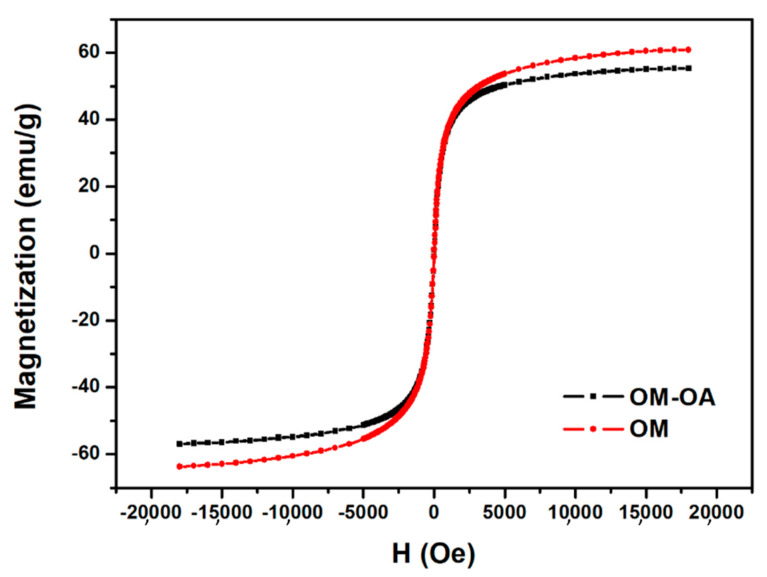
Magnetization curves as a function of the magnetic field applied at room temperature of samples OM and OM-OA.

Figure 2 shows the magnetization curve of OM and OM-OA samples as a function of the applied field at room temperature. The saturation magnetization (Ms) of the OM and OM-OA samples were 63.7 and 57.4 emu·g^−1^, respectively. The OM-OA sample shows a lower Ms value compared to the OM sample, a reduction of approximately 10.4%. This decrease can be explained by the fact that the value of this measurement corresponds to the sum of all magnetic moments present in the sample, in this case, the magnetic material (iron oxides) and the non-magnetic coating material (oleic acid). Thus, this result shows that the non-magnetic coating layer present in the OM-OA sample has a ratio of approximately 10%. In addition, the nanoparticles showed superparamagnetic behavior, since the coercivity and magnetization values of remnant are close to zero [51], corroborating the XRD results. Thus, besides the fact that the Fe_3_O_4_ nanoparticles become hydrophobic, the chemical functionalization of the magnetic oxide surfaces with oleic acid preserved the magnetic properties.

PMMA/OM-OA nanocomposites were obtained by in situ incorporation of OM-OA nanoparticles into poly(methyl methacrylate) matrices by miniemulsion technique. This polymerization process favors the formation of high molar mass lattices, narrow particle size distributions, high colloidal stability and proper encapsulation of inorganic nanoparticles [27]. All miniemulsions were prepared with 15% monomer phase (MMA) and 15% by weight OM-OA relative to monomer content. In addition, 3% by weight hexadecane and 2% initiator (BPO), both relative to monomer mass.

**Figure 3:**
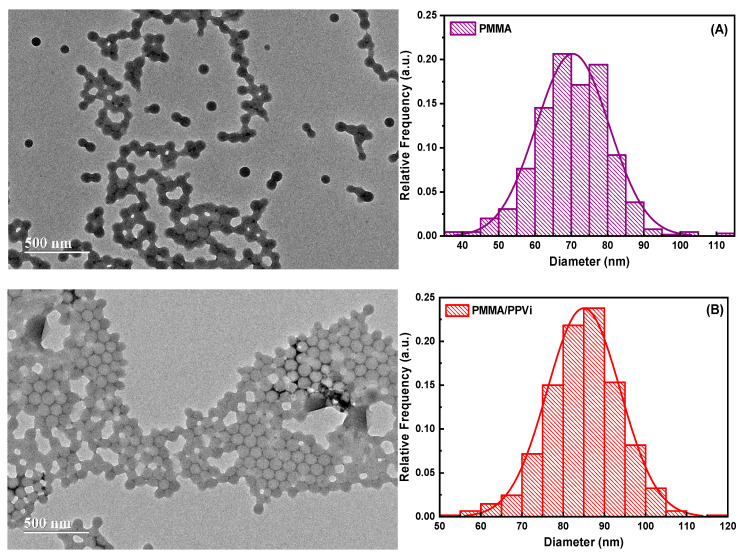
Micrographs from TEM measurements and histogram of particle size distribution (**A**) PMMA homopolymer and (**B**) PMMA/PVPi core–shell nanoparticles.

Figure 3 shows the micrographs from TEM measurements and histogram of sample size distribution (A) PMMA and (B) PMMA/PVPi. The particles showed spherical morphology and uniform size, where 326 and 307 particles were analyzed and found average sizes of 70 ± 10 nm and 85 ± 9 nm for PMMA and PMMA/PVPi respectively. Figure 3 clearly shows that a core-structure type structure was properly obtained through seeded emulsion polymerization by using precursor PMMA nanoparticles from miniemulsion prolymerization process. According to average particle size, the fraction of PVPi in the outer shell of polymer particles is expected to be in the interval from approximately 14.3 wt.% to 19.0 wt.%, which agrees very well with the theoretical PVPi mass fraction equal to 17.7% for a total consumption of the vinyl pivalate.

In the particular case of magnetic core–shell particles (PMMA/OM-OA and PMMA/OM-OA/PVPi), high conversion was achieved, around 89%, after 2 h of polymerization. Comparatively, using DLS analysis, the polymer particle diameter of the core structure obtained through miniemulsion polymerization remained virtually unchanged, exhibiting 190 nm for monomer droplets (Dg, with PdI = 0.229) and 217 nm polymer particles (Dp, with PdI = 0.254), indicating that the nucleation mechanism preferably occurs within the monomer droplets and that the coalescence and Ostwald ripening effects were minimized during polymerization (N.B. PdI values around 0.248 indicate that nanoparticles with relatively narrow distribution size are obtained). The average particles size from DLS measurements reveals an increase in the particles diameter as a result of the incorporation of Fe_3_O_4_ magnetic nanoparticles.

**Figure 4:**
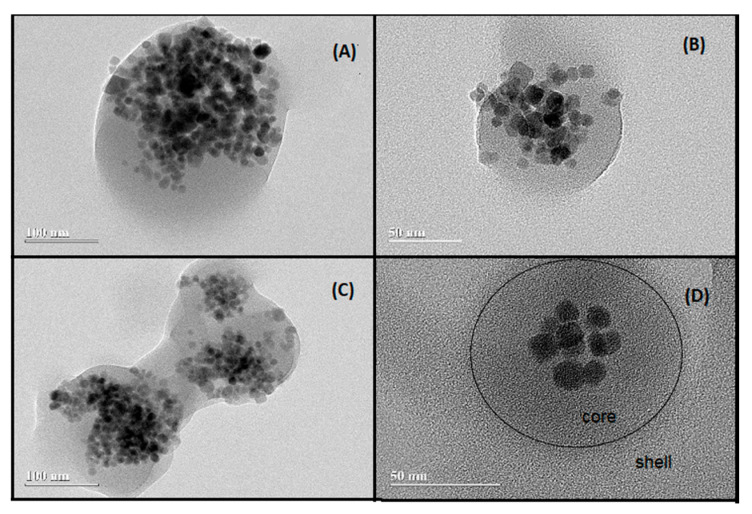
(**A**,**B**) Micrographs captured by TEM of the PMMA/OM-OA and (**C**,**D**) micrographs recorded by TEM of the core–shell poly(methyl methacrylate) nanoparticles (PMMA)/OM-OA/PVPi nanoparticles.

Figure 4 shows the TEM of the PMMA/OM-OA and PMMA/OM-OA/PVPi samples, in which it is possible to observe the presence of magnetic nanoparticles uniformly dispersed in spherical polymer nanoparticles, containing an outer layer acquiring a structure of the type core–shell. Figure 4D shows a darker part (core) and a lighter part (shell), showing the formation of a particle with core–shell morphology. Since these are polymers with a close refractive index, the TEM cannot clearly distinguish the shell and core layers. Considering the efficiency of formation of the shell structure and an average number of PMMA/OM-OA equal to 4.37·10^15^ particles in the final latex, it is expected to form the final magnetic core–shell particles with an average diameter of approximately 232 nm.

**Figure 5:**
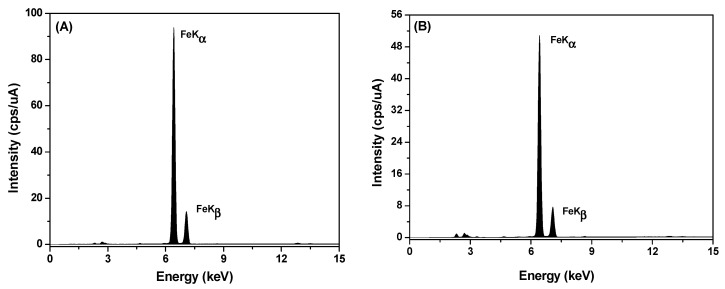
Energy dispersive X-ray spectra of the nanoparticles. (**A**) PMMA/OM-OA; (**B**) PMMA/OM-OA/PVPi.

In order to demonstrate that the nanoparticles observed are mainly composed of iron, EDX analysis was performed, being possible to observe the dominant Kα and Kβ peaks related to iron in the interval from 6.40 Kev to 7.06 Kev (Figure 5). The fraction of Fe observed in the samples was approximately 97% along with small amounts of impurities associated with Si, Ca, S, Zn, and Mn from the reagents used in the syntheses. The results observed in Figure 5 are in agreement with the experimental results described elsewhere [17,18].

**Figure 6:**
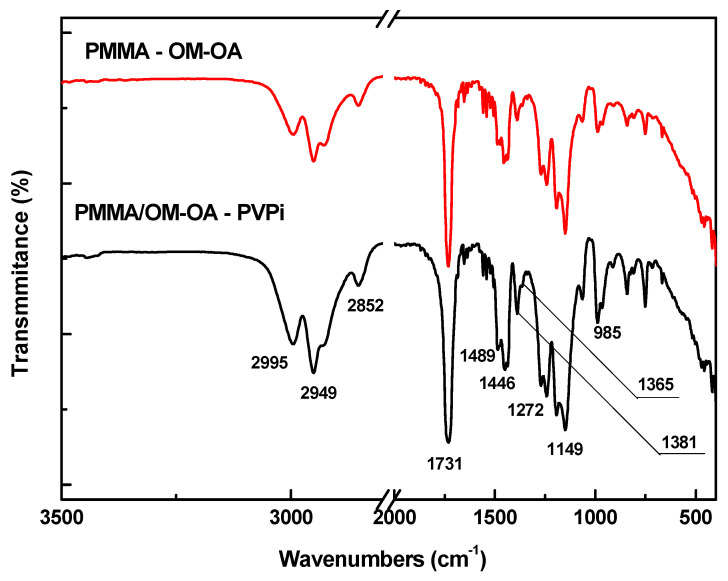
FTIR spectra of PMMA/OM-OA and PMMA/OM-OA/PVPi samples obtained from KBr pellets.

Figure 6 shows the FTIR spectrum of PMMA/OM-OA and PMMA/OM-OA/PVPi samples. The main absorption bands and their respective vibrational assignments are presented in Table 1. The absorption bands between 2995 and 2852 cm^−1^ are characteristic of asymmetrical and symmetrical stretching C-H sp^3^. The peak at 1731 cm^−1^ is attributed to the carbonyl group stretch and the 1272 and 1149 cm^−1^ bands to the C-O stretch corresponding to the ester group present in the structure of MMA, VPi, and EGDMA. Absorption at 1381 and 1365 cm^−1^ are attributed to the −CH_3_ symmetrical angular deformation of PVi tert-butyl groups [52]. The obtaining of the polymeric material is confirmed by the 1446 cm^−1^ band referring to the angular deformation of the methylene group (−CH_2_) of the polymeric chain and the absence of absorption bands between 3100 and 3000 cm^-1^ and bands between 990 and 910 cm^-1^ attributed, respectively, to the stretching and angular deformation CH sp^2^ of vinyl groups [53]. In the spectra, the presence of magnetic oxides was not confirmed due to the absence of absorption in the 578 cm^−1^ region attributed to Fe-O stretching of tetrahedral sites [54]. Nevertheless, approaching a magnet to the latex shows that the nanoparticles are attracted to the container wall. These results indicate that OM-OA are dispersed in the polymer matrix in a small amount [55].

**Figure 7:**
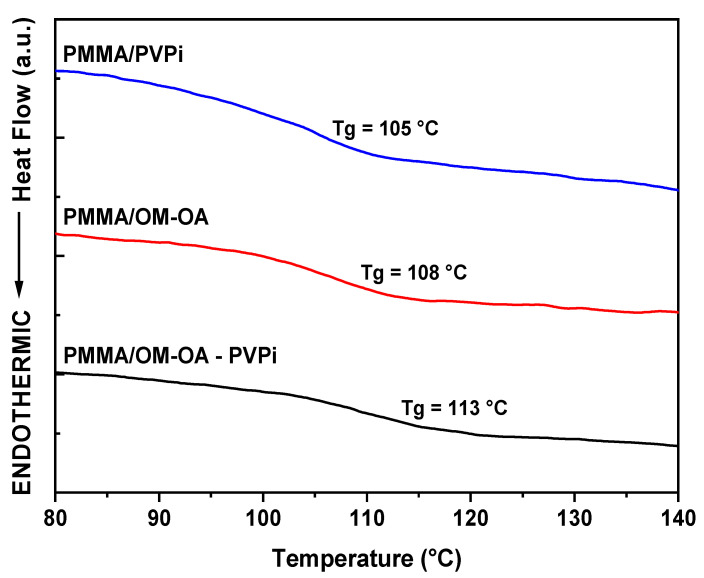
DSC curves of the PMMA/OM-OA and PMMA/OM-OA/poly(vinyl pivalate) (PVPi) samples.

The glass transition temperatures (*T*_g_) of PMMA/OM-OA and PMMA/OM-OA/PVPi samples were determined by the DSC curves shown in Figure 7. The *T*_g_ reached values greater than 110 °C, which are higher than those reported in the literature, around 104 °C [56]. This increase is attributed to the presence of OM-OA nanoparticles, restricting the mobility of the polymer chains [57] or due to a change in the tacticity of the PMMA chains [58], with major interactions between polymer chains and, consequently, increasing the *T*_g_ values. The *T*_g_ values of the PMMA/OM-OA nanocomposites were 108 °C, while those of the PMMA/OM-OA/PVPi were 113 °C. It is also worth mentioning that the increase in *T*_g_ observed here for the polymer resulted from seed emulsion process is closely related to the EGDMA used as cross-linking agent.

The mass fraction of vinyl pivalate (ℑ_*VPi*_) estimated based on the Fox model (Equation (2)) is equal to 0.16, indicating that vinyl pivalate was successfully incorporated onto the surface of the precursors PMMA particles, which explains the dominant effect of the composition on the *T*_g_ of the copolymers. It is important to emphasize that the DSC measurements corroborate the vinyl pivalate mass fraction estimated from TEM measurements, which was determined to be in the interval from approximately 0.14 to 0.19.

(2) $$\frac{1}{T_{g_{12}}}=\frac{\left(1-{ℑ}_{VPi}\right)}{T_{g_{1}}}+\frac{{ℑ}_{VPi}}{T_{g_{2}}}$$

where, $T_{g_{12}}$, $T_{g_{1}}$ and $T_{g_{2}}$ are the glass transition temperatures of the PMMA/PVPi, and in the literature of poly(methyl methacrylate) with *T*_g_ = 110 °C and poly(vinyl pivalate) with *T*_g_ = 80 °C, respectively [59,60].

**Figure 8:**
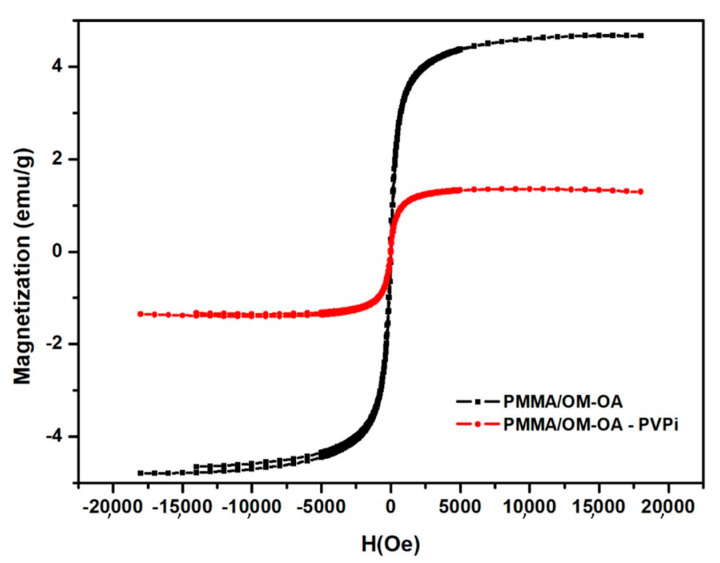
Magnetization curves as a function of the magnetic field applied to the ambient temperature of the PMMA/OM-OA and PMMA/OM-OA/PVPi samples.

The magnetic behavior of the PMMA/OM-OA and PMMA/OM-OA/PVPi samples at room temperature is shown in Figure 8. The samples showed saturation magnetization (Ms) of 4.65 emu·g^−1^ (Ms = 57.4 emu·g^−1^ of nanoparticles) and 1.35 emu·g^−1^ (56.25 emu·g^−1^ of nanoparticles), respectively. The magnetization curves have no hysteresis cycle and are reversible at room temperature, indicating that the superparamagnetic behavior of the nanoparticles was preserved during polymerization and their magnetic properties were successfully incorporated into the nanocomposites.

The Ms values of the nanocomposites were significantly lower than that of OM-OA (Ms = 57.4 emu·g^−1^), due to the existence of non-magnetic polymeric material present in the samples. Considering these Ms values, it is possible to have an estimate of the OM and OM-OA content incorporated in each nanocomposite, being, respectively, 7.3% and 8.1% for PMMA/OM-OA and 2.1% and 2.4% for PMMA/OM-OA/PVPi. In fact, these values are slightly higher, since during polymerization part of the magnetic oxides, such as Fe_3_O_4_, are converted into other iron oxides that have less magnetization, such as γ-Fe_2_O_3_, due to the presence of strong oxidants, such as BPO [17,47]. These Ms values corroborate the results of FTIR and DSC in which they show the presence of the poly (vinyl pivalate) matrix present in the nanocomposites obtained by seeded emulsion. 

**Figure 9:**
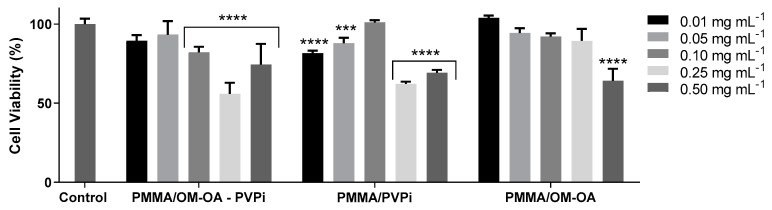
Evaluation of cytotoxic activity of poly(methyl methacrylate)/magnetic oxide coated with oleic acid/poly(vinyl pivalate)—(PMMA/OM-OA/PVPi), poly(methyl methacrylate)/poly(vinyl pivalate)—(PMMA/PVPi) and poly(methyl methacrylate)/magnetic oxide coated with oleic acid—(PMMA/OM-OA) in murine melanoma tumor cell lines, B16F10, after 24h exposure in the concentrations of 0.01 mg·mL^−1^, 0.05 mg·mL^−1^, 0.10 mg·mL^−1^, 0.25 mg·mL^−1^, and 0.50 mg·mL^−1^. The asterisks are related to the statistical significance associated with different confidence levels: * for 95% with *p* ≤ 0.05, ** 99% with *p* ≤ 0.01, *** 99.9% with *p* ≤ 0.001 and **** 99.99% with *p* ≤ 0.0001.

**Figure 10:**
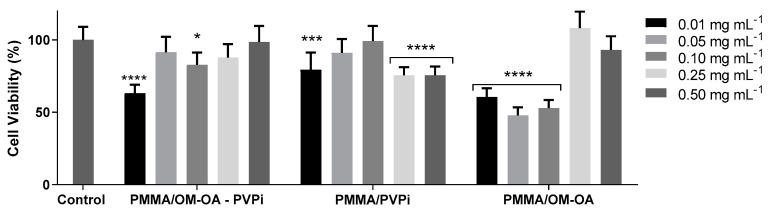
Evaluation of cytotoxic activity of poly(methyl methacrylate)/magnetic oxide coated with oleic acid/poly (vinyl pivalate)—(PMMA/OM-OA/PVPi), poly(methyl methacrylate)/poly (vinyl pivalate)—(PMMA/PVPi) and poly(methyl methacrylate)/magnetic oxide coated with oleic acid—(PMMA/OM-OA) in human keratinocyte cells, HaCaT, after 24 h in the concentrations of 0.01 mg·mL^−1^, 0.05 mg·mL^−1^, 0.10 mg·mL^−1^, 0.25 mg·mL^−1^, and 0.50 mg·mL^−1^. The asterisks are related to the statistical significance associated with different confidence levels: * for 95% with *p* ≤ 0.05, ** 99% with *p* ≤ 0.01, *** 99.9% with *p* ≤ 0.001 and **** 99.99% with *p* ≤ 0.0001.

Figure 9 and Figure 10 depict cytotoxicity results of in vitro tests performed with polymeric particles. This kind of biological test is of fundamental importance, as it is closely related to product viability, according to its level of toxicity. Considering that both human and animal cells were used, and knowing that lower percentage of cell viability, the greater its cytotoxic potential, it is necessary to evaluate influence of material concentration on cell viability in order to determine its borderline concentration.

Figure 9 illustrates performance of tested polymeric materials against murine melanoma tumor line (B16F10) in cell viability at an exposure of 24 h when compared to the control (*p* < 0.05). For each polymeric material, assays were carried out with polymer concentrations lying in interval from 0.01 mg·mL^−1^ to 0.50 mg·mL^−1^. According to Figure 9, concentration of polymer samples plays an important effect on cell viability. It is observed that cell viability is slightly affected when low polymer concentrations are employed (i.e., from 0.01 mg·mL^−1^ to 0.10 mg·mL^−1^), leading to a decrease in average cell viability of at most 18%. In general for other polymer materials, it seems reasonable to assume that borderline concentration corresponds to concentration of 0.25 mg·mL^−1^, which leads to a decrease in cell viability of approximately 45%. It is also important to bear in mind that best results were observed for core–shell magnetic polymer materials (PMMA/OM-OA and PMMA/OM-OA/PVPi), presenting a cell viability comparable to control, at concentrations of 0.01 to 0.25 mg·mL^−1^ and from 0.01 and 0.05 mg·mL^−1^, respectively. This result is very promising, considering that as an embolic agent this versatile material present a twofold action, as they can be used to both obstruct blood vessels interrupting supply of oxygen and nutrients to tumor region and hyperthermia treatment of injured area.

A similar study has been carried out for HaCaT lineage in order to evaluate the polymer materials toxicity to non-pathological cells. According to Figure 10, target magnetic polymer with core–shell structure (PMMA/OM-OA/PVPi) exhibits highest cell viability in comparison to the other tested polymer materials, and whose values of cell viability can be considered comparable and very close to control, except for concentration of 0.01 mg·mL^−1^. It is also interesting to notice that PMMA/OM-OA/PVPi performance seems not to be dependent on concentration in experimental range evaluated here. In particular case of poly(methyl methacrylate) magnetic particles (PMMA/OM-OA), high cell viability were observed for concentration of 0.25 mg·mL^−1^ and 0.50 mg·mL^−1^ equivalent to 108% and 93%, respectively.

Non-magnetic core–shell nanoparticles (PMMA/PVPi) presented an average cell viability of 84%; however, the highest cell viability very close control was approximately 99% observed for concentration of 0.10 mg·mL^−1^. 

Feuser et al. [61] evaluated in vitro cytotoxicity in murine fibroblast (L929) and normal human fibroblast (NIH-3T3) by MTT assay for 24 h of PMMA nanoparticles and nanocapsules obtained by miniemulsion polymerization. Both cells presented high cell viability, between 81 to 84% even at highest tested concentration of 750 μg·mL^−1^. Guindani et al. [62] also investigated in vitro cytotoxicity in mouse embryonic fibroblasts (NIH3T3) breast cancer (MDAMB231) and human cervical cancer (HeLa) by MTT assay for 24 h of PMMA nanoparticles and nanocapsules of PMMA conjugated with bovine serum albumin (BSA) obtained by miniemulsion polymerization at four concentrations: 100, 200, 300, and 400 μg·mL^−1^. According to authors, polymeric materials can be considered biocompatible in all concentrations tested. Mendes et al. [63] evaluated cytotoxic activity of PMMA nanoparticles obtained by miniemulsion polymerization in human leukemic cell line K562 by MTT assay for 24 h. Cytotoxic activity of polymeric nanoparticles did not induce cell death even at highest tested concentration of 1500 μg·mL^−1^.

According the Chinese standard (GB/T 16886.5-2003) materials with cell viability greater than 75% represent no cytotoxicity [64,65]. Therefore, based on the results obtained in this study, it is possible to consider that nanoparticles achieved high cell viability for both murine melanoma tumor cell lines, B16F10, and human keratinocyte cells, HaCaT., and can be considered promising for other biological studies such as in vivo tests.

## 4. Conclusions

The results showed that functionalized magnetic nanoparticles presented good dispersibility with a uniform average size of 10.5 nm, saturation magnetization of 57.4 emu·g^−1^ and superparamagnetic behavior. Miniemulsion polymerization process ensured that magnetic nanoparticles were encapsulated in polymer matrix uniformly, maintaining their superparamagnetic properties with 56.25 emu·g^−1^ of Fe_3_O_4_ nanoparticles. Results obtained are promising, since from point of view of medical application of intravascular embolization. Superparamagnetic character, essential for this medical procedure, remains present in nanocomposite and in result of seeded emulsion, in addition to presence of core–shell morphology. This new polymeric material designed to contain, as a core–shell structure, poly(methyl methacrylate)/magnetic Fe_3_O_4_/poly(vinyl pivalate), exhibited high colloidal stability, which can be linked to the desired properties of methyl methacrylate/vinyl pivalate-based polymers such as good chemical, mechanical and thermal stability.

Based on cell viability assays, the core–shell magnetic polymeric materials [a core, consisting of poly(methyl methacrylate)/Fe_3_O_4_ and a shell, formed by poly(vinyl pivalate)] can be regarded as a very promising material, as they achieved high cell viabilities for both murine melanoma tumor cell lines, B16F10, and human keratinocyte cells, HaCaT. 

## Figures and Tables

**Table 1:** Main absorption bands obtained in FTIR spectra for PMMA/OM-OA and PMMA/OM-OA/PVPi samples and their vibrational assignments.

Wavenumbers (cm^−1^)	Vibrational Assignments
2995–2852	Asymmetrical and symmetrical stretch C-H sp^3^
1731	Stretch C=O of ester
1489 and 1446	Angular deformation of methylene groups (–CH_2_)
1381 and 1365	Symmetrical angular deformation –CH_3_ of tert-butyl groups
1272 and 1149	Stretch C-O of ester
985	Out of plane angular deformation C-H
